# SI: Advances in Density Functional Theory (DFT) Studies of Solids

**DOI:** 10.3390/ma15062099

**Published:** 2022-03-12

**Authors:** Aleksandr S. Oreshonkov

**Affiliations:** 1 School of Engineering and Construction, Siberian Federal University, Krasnoyarsk 660041, Russia; oreshonkov@iph.krasn.ru; 2 Laboratory of Molecular Spectroscopy, Kirensky Institute of Physics, Federal Research Center KSC SB RAS, Krasnoyarsk 660036, Russia

This summary is a review on articles published in the Special Issue “Advances in Density Functional Theory (DFT) Studies of Solids” from the section “Materials Simulation and Design” of the MDPI journal *Materials*. Nowadays, the DFT method is widely used to calculate structural, electronic, optical, vibrational, etc., properties of materials. There is no doubt that analysis of new and practically important compounds should be carried out within the framework of theoretical (computational) and experimental methods combination. The well-established computational codes [1,2,3] based on the DFT approach were used in papers published in this Special Issue. The 7 articles discussed below [4,5,6,7,8,9,10], written by 37 authors, are excellent examples of DFT application to study various properties of solids.

The first paper written by Oreshonkov A.S. et al. [4] is aimed to characterization of structural and dynamical properties of YAl_3_(BO_3_)_4_ huntite-like borate, which is a promising material for optical applications. The electronic band structure of YAl_3_(BO_3_)_4_ is studied, and band gap values obtained using local density approximation and hybrid HSE06 functional for the first time. The importance of proper sample preparation of borate samples with high hardness for vibrational spectroscopy experiments is shown on the basis of results of DFT calculations. Bogdanov A. et al. [5] demonstrated the significant potential of the ab initio calculation of vibrational properties for identifying anionic groups and molecules in microporous natural compounds. The important information about crystal structure stability of zirconosilicates is obtained. The hypothetical structure of CaZrSi_6_O_15_·2H_2_O is predicted. In the work of Oreshonkov A.S. and Denisenko Y.G. [6] has been shown how the combination of DFT calculation of electronic properties, lattice dynamics and experimental Raman and infrared spectroscopy can be used for clarifying the structural features of rare-earth sulfates. The difference of oxygen bonds nature in Y_2_O_2_SO_4_ is discussed on the basis of partial density of states calculations. The [Y_2_O_2_]^2+^ structural units were obtained theoretically, and corresponding spectral bands were observed in vibrational spectra. Ribeiro-Claro P.J.A. et al. [7] used an inelastic neutron scattering, far infrared spectroscopy and periodic density functional calculations to study structural properties of solid-state molecular compounds. Despite the fact that internal vibrational modes of molecules were described better than external modes in the low wavenumber region, the careful inspection of this part of spectra revealed reasonable agreement between theory and experiment. Thus, matching of experimental and calculated spectra confirms the correctness of the used structural models. The excellent agreement between calculated and experimental inelastic neutron scattering spectra of crystalline 4-(dimethylamino) benzaldehyde has been obtained in the work of Nolasco M.M. [8]. The individual bands are identified and assigned to the specific structural units on the investigated compound. This fact allows us to say that leading DFT-based codes make it possible to perform an accurate interpretation of vibrational characteristics of molecular compounds in crystalline form. The article of Pankin D. et al. [9] is devoted to the problem of identification and investigation of T-2 toxin and 3-deactylcalonectrin, which can be found in such important agricultural crops as wheat and oats. The theoretical study of T-2 toxin made it possible to obtain vibrational and optical characteristics that can be used as its fingerprints. The paper by Choi Y. [10] investigates the equilibrium properties of paramagnetic hcp Fe within combination of density functional theory and alloy theory. Results of this study gives important information on hcp Fe at normal and high-pressure conditions that can be used for precise thermodynamic modeling of ferro-based alloys.
